# Synchronous Dentigerous Cysts Managed by Decompression in Non-Syndromic Pediatric Patients: Two Cases with Three-Year Follow-Up

**DOI:** 10.3390/jcm14238264

**Published:** 2025-11-21

**Authors:** Antonella Buljubasic, Dinko Martinovic, Ante Mihovilovic, Kristian Jerkovic, Ante Pojatina, Andrija Rados, Daniel Jerkovic

**Affiliations:** 1 Department of Maxillofacial Surgery, University Hospital of Split, Spinčićeva 1, 21000 Split, Croatia; alesin@kbsplit.hr (A.B.);; 2 Clinical Department of Diagnostic and Interventional Radiology, University Hospital of Split, Šoltanska 2, 21000 Split, Croatia

**Keywords:** synchronous dentigerous cysts, decompression, children, oral surgery, stomatology

## Abstract

Dentigerous cysts (DCs), usually linked to unerupted teeth, are the second most common odontogenic cysts. However, synchronous DCs are rarely seen in children without syndromic conditions. This study reports two cases of male children with no systemic illnesses who showed multiple cystic lesions in the jaw. Conventional treatment typically involves enucleation and tooth extraction, which can lead to significant complications, including infection, nerve damage that may cause temporary or permanent numbness, damage to nearby teeth, and, in cases of large cysts, jaw fractures—potentially impacting the child’s quality of life. A conservative decompression method was used, employing custom-made tubes to keep communication between the cystic and oral cavities, thereby lowering intracystic pressure. This approach resulted in complete healing of the lesions and successful eruption of permanent teeth, while safeguarding vital anatomical structures and avoiding more invasive surgery, with an uneventful clinical course. Additionally, 3-year postoperative orthopantomograms are presented, showing complete resolution of the lesions with no recurrence. These results demonstrate the effectiveness of decompression in treating multiple dentigerous cysts in pediatric patients, highlighting its advantages in preserving oral function and aesthetics while reducing surgical risks.

## 1. Introduction

Dentigerous cysts (DCs) are developmental odontogenic cysts that form around the crowns of unerupted teeth. They are the second most common type of odontogenic cysts, accounting for approximately 20–24% of all jaw cysts [1]. These cysts are typically associated with permanent teeth, particularly the mandibular third molars, followed by maxillary canines, and mandibular second premolars, so the posterior mandible (46.3%) and anterior maxilla are the most common predilection sites (27.8%) [2,3]. Although DCs predominantly affect males more than females, with an approximate ratio of 1.6:1, they are most commonly diagnosed in individuals between the second and fourth decades of life [2,3]. Compared to adults, DCs are relatively rare in children, with a prevalence rate of 1.44 per 100 unerupted teeth, and only 9% of DCs occur in the first decade of life [4].

From a histopathological perspective, dentigerous cysts typically present a non-keratinized stratified squamous epithelium with occasional elongated, interconnecting rete ridges. A variable number of chronic inflammatory cells may be observed in the underlying connective tissue. There are several theories that attempt to explain cystic growth. The widespread intrafollicular theory states that fluid accumulation occurs between the reduced enamel epithelium and the crown of an unerupted tooth, often due to pressure from the impacted tooth or inflammation. Other theories suggest a developmental origin from the tooth follicle itself or that inflammation from a nonvital deciduous tooth spreads to the follicle of its permanent successor. Once formed, the cyst’s growth is driven by the accumulation of fluid and the pressure exerted on the surrounding bone [3].

Multiple DCs are extremely rare and are primarily associated with syndromes such as Gorlin–Goltz, Gardner, Mariteaux–Lamy, Klippel–Feil syndrome, and Cleidocranial dysplasia [5,6,7]. The traditional treatment for DCs involves surgical enucleation combined with the extraction of the associated unerupted tooth. However, this approach can lead to loss of permanent teeth and necessitate prosthetic rehabilitation. Complications include infection, nerve damage that may result in permanent or temporary numbness, damage to adjacent teeth, and, in significantly large cystic lesions, jaw fractures [8,9,10,11,12]. An alternative method is decompression or marsupialization, which involves creating a communication between the cystic cavity and the oral environment to reduce intracystic pressure. This technique is less invasive, preserves vital structures, and allows spontaneous eruption of teeth. Decompression is particularly advantageous in pediatric patients due to its minimally invasive nature. It involves maintaining an opening in the cyst wall using custom-made tubes or obturators. Various devices have been used by clinicians, including intravenous catheters, plastic cannulas, individual acrylic stents, and even dental suction secured with sutures, wires, or fixation screws. Complications such as tube dislodgement or mucosal irritation can occur but are generally manageable with proper follow-up care [9,10]. We modified the surgical tube commonly used for hepatobiliary drainage in both cases. The device was easy to apply and adjust, comfortable to wear, simply irrigated, did not cause inflammation or irritation of the mucosa, and had adequate retention [11,12].

We present two cases of non-syndromic pediatric patients with synchronous dentigerous cysts treated by decompression with a custom-made tube. These are the first case reports showing the complete resolution of multiple cystic lesions in children treated only by decompression. Three-year postoperative radiographs are also presented.

## 2. Case Presentation

### 2.1. Case 1

A 10-year-old boy was referred to the Department of Oral and Maxillofacial Surgery because routine orthopantomography revealed unicystic radiolucency in the maxilla and mandible. On the left side of the mandible, the buccal cortical was thin and exhibited bone elasticity on palpation. Clinically, the maxilla showed no signs of pathology. The patient denied a sensory deficit. Systemic disease, previous traumatic injury, or use of medications was not noted.

**Figure 1:**
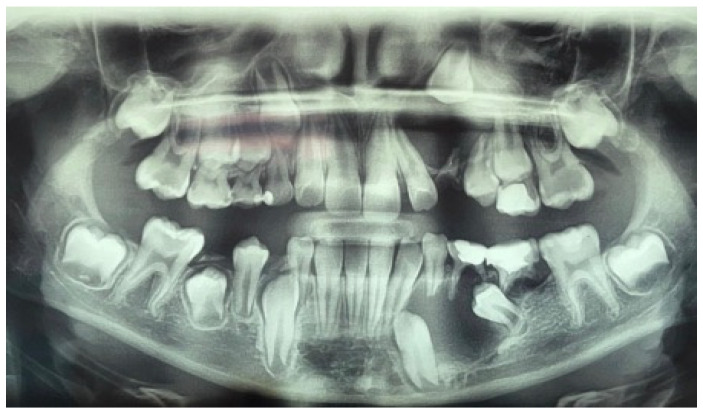
Preoperative panoramic radiograph of a ten-year-old boy showing unicystic radiolucencies on the left side of the maxilla and mandible.

A panoramic radiograph and cone beam computed tomography (CBCT) showed a well-defined unicystic radiolucency extending from the left maxillary second incisor to the primary second molar on the same side. The permanent canine was impacted toward the maxillary sinus, and the root of the second incisor was tilted mesially by the lesion. Another radiolucency extended from the left lower lateral incisor to the primary second molar. Hypodontia of the permanent premolar was noted. The lesion was in contact with the inferior alveolar nerve [Figure 1].

Based on these findings, a provisional diagnosis of a dentigerous maxillary and mandibular cyst was made. The primary left maxillary canine, the second primary maxillary molar, the primary left mandibular canine, and the first and second molars were extracted under general anesthesia. A biopsy was taken at both sites for histopathological examination. Decompression devices were made out of surgical tubes used for hepatobiliary drainage in abdominal surgery (T-FR Huali Technology, Changchun, China). They were first cut to the desired length, as measured on the preoperative CBCT scan, to fit the cystic lumen. Then, their horizontal parts were adjusted, creating “wings” that enabled easier fixation onto the mucosa, and they were secured with 4-0 nylon sutures.

The patient’s parents were instructed to irrigate the cyst cavities with 0.9% saline three times daily. Follow-up appointments were scheduled every three months after surgery. Histopathologic examination confirmed DCs, showing an inflamed fibrous wall lined with squamous epithelium.

**Figure 2:**
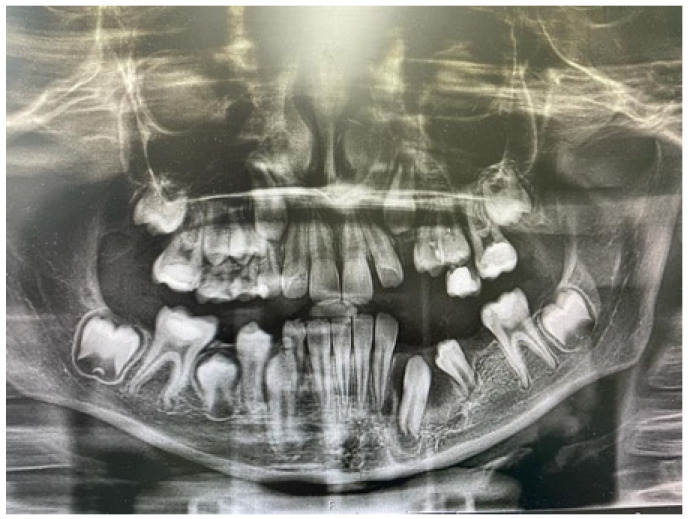
Three-month postoperative panoramic radiograph showing a significant reduction of the lesions. The decompression tubes were removed.

Three months later, the postoperative radiograph showed significant regression of the lesions in the maxilla and mandible, respectively. In addition, the maxillary canine was more extruded, and the mandibular canine and first premolar were more vertically positioned [Figure 2].

**Figure 3:**
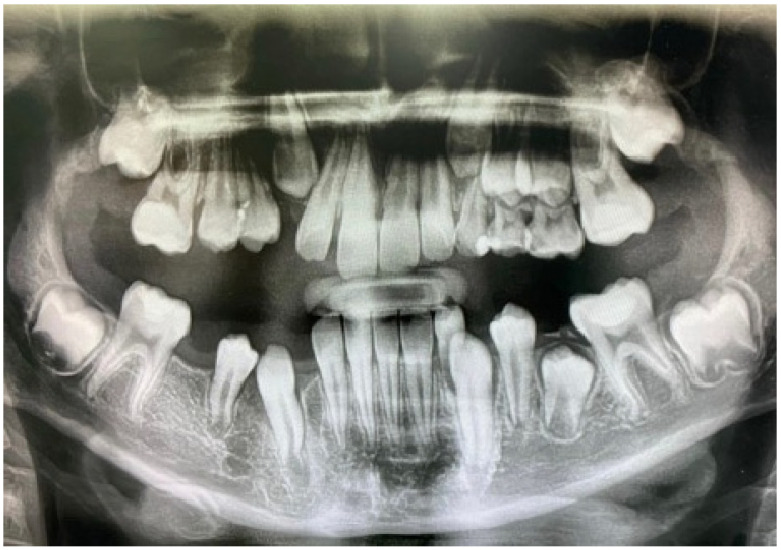
Twelve-month postoperative panoramic radiograph showing the spontaneous eruption of left maxillary canine and mandibular left canine and first premolar with resolution of both lesions.

**Figure 4:**
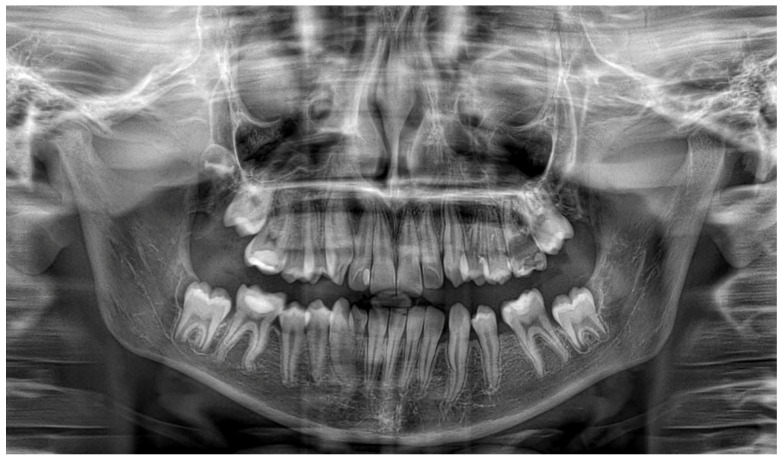
A three-year follow-up panoramic radiograph showing full resolution of the pathologies.

One year after decompression, all the permanent teeth involved in the processes erupted, preserving pulpal vitality. The decompression tube had to be adjusted due to the eruption of the canine. Complete regression of both lesions with bone deposition was observed radiographically [Figure 3], and the left inferior alveolar nerve innervation was preserved. A three-year follow-up panoramic X-ray shows no recurrence of the cystic lesions [Figure 4]. Unfortunately, the cavity caused severe destruction of the left permanent first molar.

### 2.2. Case 2

A 9-year-old boy with a non-significant medical history was referred to our department because of a painless swelling on the right side of the mandible. Systemic disease, previous traumatic injury, or use of medications was denied.

**Figure 5:**
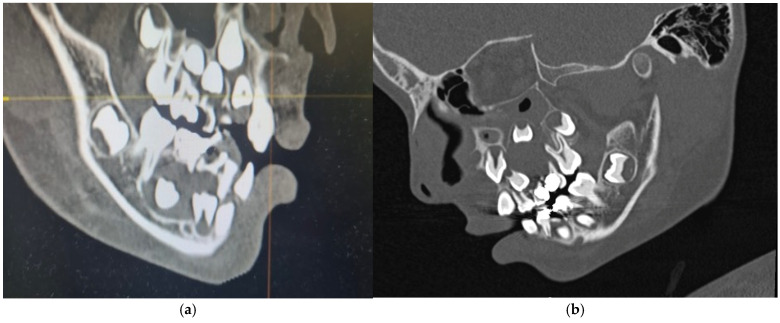
Preoperative MSCT of a nine-year-old boy showing unicystic radiolucencies on the right side of the mandible (**a**) and left side of the maxilla (**b**).

The orthopantomogram, brought by the patient, presented unycistic radiolucency with well-defined margins on the mandible’s right side as well as the left side of the maxilla. A multi-slice computed tomogram (MSCT) showed a cystic lesion in the maxilla extending from the second left permanent incisor to the first permanent molar. It included the unerupted follicles of the permanent canine, first, and second premolar, and was separated from the maxillary sinus by a thin bone. A second lesion was observed in the mandible, extending from the primary right canine to the first permanent molar. It also involved the crowns of the unerupted permanent teeth [Figure 5a,b].

**Figure 6:**
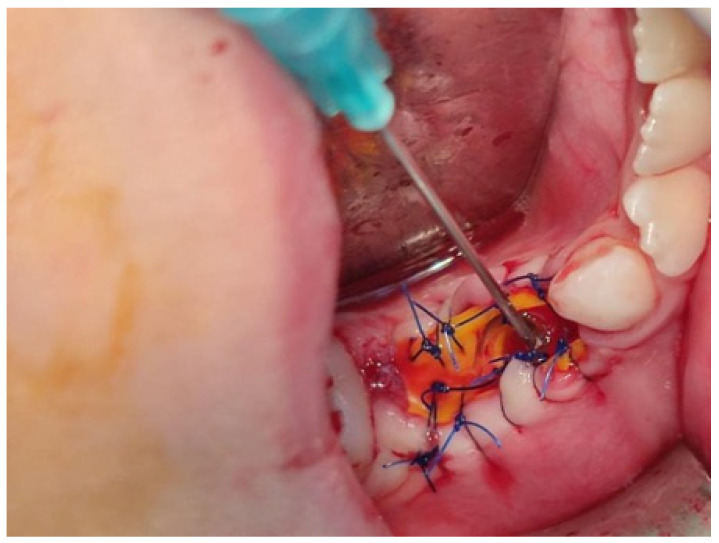
Decompression tube in place with needle for daily irrigation with saline solution.

**Figure 7:**
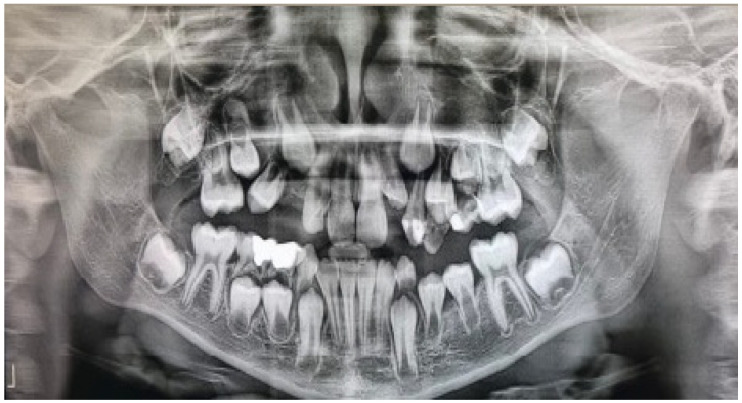
Twelve-month postoperative panoramic radiograph showing the resolution of both lesions.

**Figure 8:**
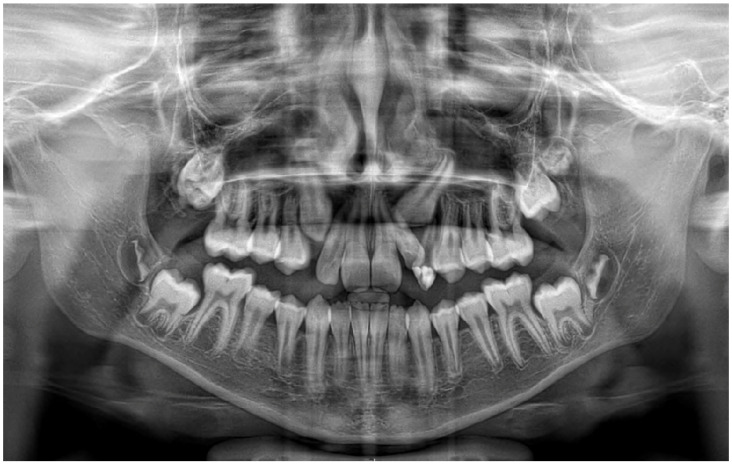
A three-year follow-up panoramic radiograph showing no recurrence of the lesions.

The same procedure was performed as in the first case, in which the first and second upper and lower deciduous molars were extracted. A biopsy was taken at both sites, and the same decompression devices were placed in the extraction sockets. The patient was advised to irrigate the cyst cavities with saline [Figure 6]. Histopathologic analysis of the lesions confirmed the diagnosis of dentigerous cysts, characterized by lining of 2–4 layers of non-keratinized epithelium and infiltration of inflammatory cells within the fibrous wall. Postoperative radiographs showed a gradual reduction of the lesions, and all permanent teeth erupted after one year [Figure 7]. No recurrence of the lesions was noted on a three-year follow-up radiograph [Figure 8]. The patient was referred to an orthodontist for the correction of occlusion.

## 3. Discussion

Multiple dentigerous cysts are rare in healthy children, with only 17 cases identified from 1943 to 2005 [13]. Multiple jaw cysts are a major feature of Gorlin–Goltz syndrome, affecting 90% of cases, along with basal cell carcinomas, epidermal cysts, pits, enlarged head, and ocular hypertelorism. Histologically, these cysts are odontogenic keratocysts (OKC) with high recurrence, treated similarly to dentigerous cysts: enucleation for small cysts, decompression or marsupialization for larger ones. Radiographic discovery of multiple cysts warrants evaluation for Gorlin–Goltz syndrome [14,15,16,17].

When treating DCs, especially in children, choosing between decompression and enucleation with extraction involves weighing the benefits and drawbacks of each method. Published research shows that many DCs are primarily managed through enucleation and extraction of permanent teeth [2]. This procedure involves the complete surgical removal of the cyst along with any associated unerupted teeth and is considered definitive, offering complete resolution of the cystic lesion and lowering the risk of recurrence. Additionally, it allows for thorough histopathological analysis, which helps in accurate diagnosis and management. However, as mentioned earlier, it is more invasive than decompression and can lead to greater surgical morbidity, including potential damage to nearby structures such as nerves or tooth roots. Furthermore, it often requires the removal of affected unerupted teeth, which can impact the dental arch’s integrity and may necessitate extensive prosthetic rehabilitation.

On the other hand, decompression is a more conservative technique that involves creating an opening in the cyst to allow continuous drainage and reduce intracystic pressure. This approach is minimally invasive and aims to preserve vital anatomical structures and promote the spontaneous eruption of impacted teeth. It is particularly beneficial in children because it maintains dental arch integrity and minimizes surgical morbidity. Decompression helps preserve permanent teeth and pulp vitality, reducing the need for prosthetic rehabilitation. When used alongside orthodontic treatment, it helps reposition impacted teeth and aids their development. Key factors include age (under 10 years), the depth of impaction, and germ angulation (less than 25°) [8]. However, it requires patient compliance with postoperative care, such as regular irrigation of the cyst cavity, and may involve a prolonged treatment period with frequent follow-up visits. Therefore, decompression offers a less invasive alternative that aligns well with pediatric dentistry goals, such as preserving natural dentition and supporting normal oral development [18]. Common complications include the insertion or loss of a decompression tube, irritation, and hypertrophy of the oral mucosa [9].

Published research shows that many DCs are mainly treated through enucleation and removal of permanent teeth [2]. An 18-year retrospective review of dentigerous cyst treatment in children found that about 72% of patients needed at least one permanent tooth extraction during surgery, mostly in the mandibular posterior region [19]. Conversely, a 10-year retrospective study by Marin et al. revealed that 72% of dentigerous cysts resolved with a single decompression, thus avoiding secondary enucleation. This aligns with a systematic review by Cobo-Vázquez et al., which emphasizes conservative treatment as a reliable procedure with an 83% success rate for marsupialization and 100% after decompression. Both studies recommend decompression as the first approach for larger cysts, especially in children. Factors such as histological differences (e.g., dentigerous cyst versus keratocyst), maintaining an open cystic lumen, and younger age influence the success of decompression [10,20].

Most reports on this topic in scientific literature are case studies, including ours, leading to a shortage of comprehensive scientific data and consensus on the best surgical approach for cystic lesions in children. There is a necessity for multi-center collaboration and standardized treatment guidelines for this group.

## 4. Conclusions

Synchronous DCs not linked to any syndrome or systemic disease are a rare finding and thus require special mention. If feasible, aggressive surgical procedures should be avoided in such cases, especially in pediatric patients. Decompression can be an effective treatment option because it helps preserve essential structures and promotes the spontaneous eruption of permanent teeth, thereby reducing the need for extensive prosthetic rehabilitation. A cooperative patient, along with histopathological examination and regular follow-up appointments, is essential for successful treatment outcomes.

## Data Availability

The original contributions presented in this study are included in the article. Further inquiries can be directed to the corresponding author.

## Abbreviations

The following abbreviations are used in this manuscript:

DC	Dentigerous cyst
MSCT	Multi-slice computed tomogram
OKC	Odontogenic keratocyst
CBCT	Cone beam computed tomography
